# Research on the Integration of Cultural Tourism Industry Driven by Digital Economy in the Context of COVID-19—Based on the Data of 31 Chinese Provinces

**DOI:** 10.3389/fpubh.2022.780476

**Published:** 2022-03-09

**Authors:** Xiangyin Li, Xueping Liang, Ting Yu, Sijia Ruan, Rui Fan

**Affiliations:** ^1^School of Chinese Language and Culture, Zhejiang Yuexiu University, Shaoxing, China; ^2^Shaoxing Culture and Tourism Industry Research Institute, Zhejiang Yuexiu University, Shaoxing, China; ^3^School of Economics, Tianjin University of Commerce, Tianjin, China; ^4^Institute of International Education, New Era University College, Kajang, Malaysia

**Keywords:** digital economy, cultural tourism, COVID-19, China, integration

## Abstract

This article examines the impact of digital economy on the integration of China's cultural tourism industry in the context of COVID-19 by measuring the integration degree of cultural tourism industry as a substitute variable of cultural tourism integration. The empirical study found that the development of digital economy during the COVID-19 pandemic did promote the integration of China's cultural tourism industry, and compared with year 2019, the digital economy has strengthened the integration of cultural tourism industry. During the COVID-19 pandemic, the development of digital economy has promoted the integration of China's cultural tourism industry, and the positive effect of digital economy on the integration of China's cultural tourism industry has gradually strengthened compared to previous ones. The digital economy has played a mediating role in the impact of COVID-19 on the integration of China's cultural tourism industry. Therefore, China should formulate macropolicies and digital economy-related policies to strengthen the ability of digital economy to deal with risks and improve the digital system.

## Research Background and Literature Review

COVID-19 broke out in Wuhan in December 2019 and spread across the country at a breakneck speed. As of August 29, 2021, the number of confirmed cases in China has reached 151,122,891. The outbreak of COVID-19 has brought a huge impact on the cultural and tourism industry, especially the film industry, tourism industry, travel agencies, entertainment industry, cruise industry, and tourism transportation. With the rapid spread of COVID-19, China's overall loss scale may be stronger than that of SARS, but the loss proportion is smaller, mainly due to the emergence of the digital economy, which provides a new way for the integration of cultural tourism industry (1).

### Integration of Cultural Tourism Industry

At present, scholars all over the world have different emphases on cultural tourism industry integration. Foreign scholars pay more attention to the choice of the integration path of the cultural and tourism industry. Addo (2) stressed the importance and necessity of European heritage and cultural diversity for Ghana's tourism industry. To promote the economic development of domestic tourism, tourism facilities should be improved and products should be diversified through innovation, so as to integrate cultural industry with tourism industry. Through factor analysis and reliability test, Hung (3) and other foreign scholars extracted five motivation factors, namely novelty, exploration, family reunion, restoration of balance, and socialization, to study the motivation of festival tourists and put forward that festival has become one of the fastest growing tourism modes. The research of Ling (4) shows that festival events are increasingly becoming an important tool for economic development, not only through tourism, but also by rebranding cities and regions as modern cultural centers. Krakowiak (2014) introduced the potential and importance of Museums in Poland in cultural tourism, which plays an important role in driving the tourism economy. Juzefovič (5) studied creative tourism from the perspective of philosophy, sociology, and communication and proposed that innovative tourism is a new generation of tourism. This new type of tourism no longer manipulates and develops cultural, personal, and natural resources such as traditional tourism, but adds value and enriches cultural, personal, and natural resources.

The research of domestic scholars on the integration of cultural tourism industry mainly focuses on the relationship, integration mechanism, and integration barriers among cultural tourism industries. Taking Yichang as the research object, Cao et al. (6) deeply analyzed the mutual promotion and integration of cultural tourism industries. Zhao (7) believes tourism and culture complement each other. The openness of tourism enables it to receive cultural indoctrination, and the permeability of culture enables it to transcend the boundary of industry and establish integration with tourism. This, Bao and Wang (8), Zhang (9), Feng (10), and Li and Weng (11), used quantitative methods to quantitatively study the integration relationship between cultural industries using methods such as coupling coordination degree and Herfindahl index method. Zhang (12) believes that external environment is the main reason driving the integration of cultural and tourism industry, such as diplomatic relations, government policies, and consumer's demand. Zhao (7) believes that the inherent characteristics of cultural industry and tourism, such as the openness and permeability of the tourism industry, are the main driving force for the integration of the cultural and tourism industry and the internal driving force for the sustainable development of the cultural and tourism industry. In addition, Wang and Wu (13) proposed in his research that the growth of the population's demand for spiritual civilization is the fundamental driving force for the comprehensive development of cultural tourism industry.

Zhuang (14) qualitatively analyzed the driving effect of cultural and tourism industry integration on regional economic development, social progress, and construction of spiritual civilization. Shi (15) believes that the integration of cultural and tourism industry deepens the connotation of tourism on the one hand, expands the market of cultural industry on the other hand, and provides impetus for the development of other related industries. Huo (16) put forward four positive effects in her research: the integration of cultural tourism industry will improve the independent innovation ability of the region, strengthen market competitiveness, drive consumer demand, and facilitate regional integration. However, Shi (17) found in the process of studying the integration of Sichuan's cultural and tourism industry that the integration of cultural tourism industry has great advantages. On the one hand, it can enrich cultural tourism products and open up new markets. On the other hand, it can save the operation cost of the two major industries, realize the value-added of the industrial chain, and bring innovation to the organization and management.

### Integration of COVID-19 and Cultural Tourism Industry

Cui (18) believes that COVID-19 has a great impact on the cultural tourism industry, but it is not a devastating blow. The integration of cultural and tourism industry has the following advantages: consumption advantage, industrial advantage, and economic advantage. The consumption of culture and tourism has become the basic needs of the people; In addition, the productivity system of the cultural tourism industry has not been seriously damaged in the COVID-19 pandemic. Service facilities such as hotels, B&Bs, scenic spots, large restaurants, tourism transportation facilities, museums, tourism performances, cultural and creative industries, cinemas, and online tourism have not disappeared, and even the productivity level has not been substantially reduced. Our economy is now on the upswing, and consumers have plenty of spending power, and obviously, we can expect that once the epidemic disappears, these stockpiles of physical and psychological demand will rebound strongly. If not all of the damage, at least a large part of our economy will be restored. Deng et al. (19) and others conducted in-depth analysis of the impact of the epidemic on the cultural tourism industry from the perspectives of demand and supply. To find a breakthrough for the revitalization of the cultural tourism industry, they proposed that for the revitalization of the cultural tourism industry after the epidemic, government support at the macrolevel, industrial linkage at the medium level, and enterprise development at the microlevel should be combined for coordinated development. Yuan's (20) research shows that the impact of COVID-19 on the cultural and tourism industry varies in different periods. In the short term, the impact of COVID-19 will continue for some time. The state's support for medium, small, and microcultural and tourism enterprises should gradually shift from simple financial subsidies to reducing operating costs, so as to ensure the operation of medium, small, and microcultural and tourism enterprises and avoid layoffs to cut costs, resulting in a large number of unemployment. In the medium term, the government can promote government-enterprise cooperation. Cultural and tourism enterprises can develop digital content products. The government can establish a “cultural cloud” platform for integration and then connect with professional publicity companies to achieve a win–win situation. In the long run, cultural competitiveness can be enhanced by building representative cultural brands. This shows that the government has taken various measures to revitalize the cultural and tourism industry, which has been hit hard by the epidemic.

Chinese scholars have also put forward different policy recommendations for the recovery of the cultural tourism industry after COVID-19. Yao (21) believes that first of all, we should reduce taxes and fees for the cultural tourism industry, increase investment and capital introduction, enhance the transformation of characteristic culture and service infrastructure in scenic spots, and enhance project construction. It is also pointed out that the ideological transformation of people and the digital transformation of various industries during the epidemic period provide an opportunity for the integration of cultural tourism industry. Jiang et al. (22) proposed that the government should encourage the joint operation of cultural and tourism industry to increase the ability to resist risks. At the same time, it is also necessary to promote the construction of “5G+ smart tourism,” build a smart cultural tourism ecosystem, promote innovation with technology, focus on the integration of “science and technology + cultural tourism,” accelerate the digital transformation of the cultural and tourism industry based on 5G and artificial intelligence new technology, create diversified cultural and tourism products, and improve the supply structure of cultural and tourism. Zhong (23) proposed that the way to promote the integration of culture and tourism through culture and tourism consumption is to implement appropriate subsidies for consumption, tax and fee reduction, and other measures. The second way is through the digital economy to promote industrial upgrading, cultural tourism development at the same time in the form of “big data + consumption subsidy,” so as to promote to build big platform—“brigade cloud” data analysis and decision system, set up including consumer's attributes, big data, such as position and consumer's preferences to achieve precise subsidies, and also play a multiplier effect on consumption.

Based on the above analysis, this article proposes hypotheses 1 and 2:

H1:COVID-19 has negatively impacted the integration of China's cultural tourism industry in the short term
H2:COVID-19 has had a long-term positive impact on the integration of China's cultural tourism industry

### Integration of Digital Economy and Cultural Tourism Industry

The impact of COVID-19 on China's economy is huge, and the impact on China's cultural tourism industry is also significant. However, with the development of modern information technologies such as 5G, artificial intelligence, and big data, digital economy provides a new way for the integration of China's economy and cultural tourism industry. Xia (24) shows that with the digital transformation of the industry and the improvement in public service and policy system, the cultural and tourism industry will achieve in-depth integration in a wider range, deeper level, and at a higher level, which will also bring about the transformation of the development mode of the cultural and tourism industry and the development of new formats. Deng et al. (19) believes that the cultural and tourism industry has been greatly impacted by the epidemic. However, in the context of the rapid development of information technology, the continuous transformation of cultural and tourism industry and the rapid change of tourism market demand, to promote the high-quality development of the industry, we should seize the opportunity of the development of digital economy and constantly improve their digital, network, and intelligent development. Cai and Ding (25) proposed the integration model of digital economy + cultural and tourism industry based on the investigation of Chengdu. They believe that the digital economy is not only a factor for the transformation and upgrading of the cultural and tourism industry and the elimination of backward production capacity, but also an ecological environment for the future development of the cultural and tourism industry. It puts forward that we should make good preparations at macro-, medium, and microlevels to promote the deep integration of China's cultural and tourism industry and digital economy. At the macrolevel, we should improve the policy system and do a good job in the top-level design; at the medium level, we should strengthen the market subjects and improve the investment and financing channels; at the microlevel, we should optimize the talent structure and strengthen the integration of resources.

Hojeghan (26) believes that digital economy is no longer limited to commercial trade and services, and the development of digital economy affects various aspects of life, from health to education, from commerce to banking. They propose that digital economy promotes the integration of cultural tourism industry through technological integration. The research of Voronkova (27) focuses on the new direction of the digital transformation of tourism. Under the impact of COVID-19, consumers are unable to travel and the demand of the tourism industry is seriously insufficient. Tourism digital transformation into virtual tourism can effectively deal with the impact of emergencies. Stelnik (28) believes that the development of digital economy will greatly improve the efficiency of tourism management and guide the development of tourism enterprises. Bozhuk (29) studied the current situation of China's tourism industry from the perspective of consumer's behavior. The development of digital economy can effectively identify consumer's needs, thus determining the main direction of tourism transformation under the digital economy.

Based on the above analysis, this article proposes hypothesis 3:

H3:COVID-19 has had a positive impact on the integration of the cultural tourism industry in the long term through the development of the digital economy

Based on previous studies on the level of integration of COVID-19, digital economy, and cultural tourism industry, scholars generally focused on theoretical research and policy suggestions, and few empirical studies were conducted to explore the connection among the three. Therefore, this article takes 31 provinces of China as the research object to study the impact of digital economy on the integration of China's cultural tourism industry in the context of COVID-19.

## Measurement of Cultural Tourism Industry Integration

At present, there has not been a unified conclusion on the measurement method of industrial convergence in academia. Some scholars use the Herfindahl index method to measure industrial convergence. In the technology of the Herfindahl index method, the correlation coefficient of technology patents is used to measure, which has great advantages in measuring technology integration. However, at present, there are few patent data related to the cultural tourism industry, and it is difficult to make statistics. Therefore, this article introduces the coupling coordination degree model of cultural tourism industry integration. Since different indicators have different contributions to cultural industry and tourism, this study adopts linear weighting method to comprehensively evaluate cultural industry and tourism, and its formula is as follows


(1) $$\begin{array}{r} u_{i}=\overset{n}{\underset{j=1}{\sum }}w_{ij}u_{ij},\overset{n}{\underset{j=1}{\sum }}w_{ij}=1 \end{array}$$

*u*_*i*_ represents the comprehensive evaluation value of industry, *u*_*ij*_ represents the contribution of indicator J to industry i in this industry, *w*_*ij*_ is the weight of index j in system i, and the weight is determined by entropy method.

when *u*_*ij*_ is a positive indicator:


(2) $$\begin{array}{r} u_{ij}=\left(X_{ij}-X_{j\min}\right)/\left(X_{j\max}-X_{j\min}\right) \end{array}$$

when *u*_*ij*_ is a negative indicator:


(3) $$\begin{array}{r} u_{ij}=\left(X_{j\max}-X_{ij}\right)/\left(X_{j\max}-X_{j\min}\right) \end{array}$$

### Coupling Coordination Degree Evaluation Model

The degree of coupling coordination is used to measure the degree of integration between cultural industry and tourism. The coupling coordination model is used to calculate the coupling coordination scores of two industries. This is helpful to analyze the coordination level of different industries and provide a theoretical basis for promoting the integrated development of culture and tourism industry. When calculating the interaction between the two industries, the coupling degree is:


(4) $$\begin{array}{r} C=2{\left(u_{1},u_{2}\right)}^{1/2}/\Pi \left(u_{1}+u_{2}\right) \end{array}$$

where C represents the coupling coordination degree of cultural industry and tourism industry, *u*_1_, *u*_2_ represent the comprehensive evaluation scores of the two industries, respectively. Coupling of the two industry systems:


(5) $$\begin{array}{r} P=\alpha u_{1}+\beta u_{2} \end{array}$$

(6) $$\begin{array}{r} D=C\times P \end{array}$$

D represents the coupling coordination degree of the two industries, and P represents the evaluation index of the comprehensive development level of the two industries. To directly reflect the coupling coordination degree between the cultural industry system and the tourism industry system, this article classifies *p*-values according to Liao Chongbin and analyzes the coupling coordination level of various provinces and autonomous regions in China on this basis. See Table 1

**Table 1:** Division of coupling coordination level.

**Coupling coordination**	**Coordination level**	**Coupling coordination**	**Coordination level**
0.00–0.09	Extreme imbalance	0.10–0.19	Serious imbalance
0.20–0.29	Moderate disorders	0.30–0.39	Mild disorder
0.40–0.49	On the verge of disorder	0.50–0.59	Barely coordination
0.60–0.69	Primary coordination	0.70–0.79	Intermediate coordination
0.80–0.89	Good coordination	0.90–1.00	Good coordination

### Index Selection

In the construction of index system, cultural industry and tourism industry cannot be completely separated. Otherwise, it is impossible to give a good explanation of industrial integration. Therefore, in the construction of the index system, we should fully consider the integration of cultural tourism industry and the characteristics of the two industries, and based on the availability of data, consider the industrial scale, industrial benefits, and industrial elements. See Table 2 for details.

**Table 2:** Evaluation index system of cultural industry and tourism industry development level.

**System**	**First–level indicators**	**Second-level indicators**
		Cultural expenses (ten thousand Yuan)
		Total operating revenue of cultural market (ten thousand Yuan)
	Industrial scale	Number of museum visitors (ten thousand/person)
		Audience of arts group performance (ten thousand/person)
		Operating income of cultural service Industry (ten thousand Yuan)
Culture industry	Industrial benefit	Operating income of cultural manufacturing industry (ten thousand Yuan)
		Operating income of wholesale and retail enterprises (ten thousand Yuan)
		Art performance group performance income (ten thousand Yuan)
		Number of museums (units)
		Number of public library institutions (units)
	Industry factors	Number of performing arts venues (units)
		Number of legal entities in cultural and related industries (units)
		Employees of cultural institutions (ten thousand people)
		Domestic tourism revenue (100 million yuan)
	Industrial scale	Foreign exchange income from tourism (USD 100 million)
		Domestic tourist number (ten thousand/person)
		Inbound tourists (ten thousand/person)
		Operating income of travel agency (ten thousand Yuan)
Tourist industry	Industrial benefit	Star hotel operating income (ten thousand Yuan)
		Operating income of tourist attractions (ten thousand Yuan)
		Number of travel agencies
	Industry factors	Number of star-rated hotels
		Number of tourist attractions
		Tourism industry employees (ten thousand people)

The weight of each index is calculated by entropy method in this article. The steps of entropy method are as follows:

(1) Construct the original evaluation matrix: Suppose there are t years, A provinces, and B evaluation indexes, then the original evaluation matrix is *X* = (*x*_θ*ij*_). J = 1,..., b, for example, (*x*_θ*ij*_) in the matrix represents the j^th^ index value of province i in year θ in this system.
(2) Dimensionless processing is carried out for indicators. For positive indicators, formula 7 is adopted, whereas for negative indicators, Formula 8 is adopted. To avoid the occurrence of zero value, each indicator is translated by 0.1 unit after standardization in this article.
  (7) $$\begin{array}{r} M_{\theta ij}=\frac{x_{\theta ij}-\min_{j}\left(x_{\theta ij}\right)}{\max_{j}\left(x_{\theta ij}\right)-\min_{j}\left(x_{\theta ij}\right)}+0.1 \end{array}$$
  (8) $$\begin{array}{r} M_{\theta ij}=\frac{\max_{j}\left(x_{\theta ij}\right)-x_{\theta ij}}{\max_{j}\left(x_{\theta ij}\right)-\min_{j}\left(x_{\theta ij}\right)}+0.1 \end{array}$$
(3) Calculate the proportion of each indicator in the corresponding sample, as shown in Formula 9
  (9) $$\begin{array}{r} p_{\theta ij}=\frac{M_{\theta ij}}{\underset{j}{\sum }M_{\theta ij}} \end{array}$$
(4) Calculate the entropy value and difference index of the index, as shown in Formula 10
  (10) $$\begin{array}{r} e_{j}=k\underset{j}{\sum }p_{\theta ij}\ln\;\left(p_{\theta ij}\right),\;g_{i}=1-e_{i} \end{array}$$
  $k=\frac{-1}{\ln\;\left(nt\right)}$, Where n is the number of research samples and t is the year interval.
(5) Calculate the weight of each indicator and calculate the comprehensive score, as shown in Formula 11, where *w*_*j*_ is the weight of indicator j,
  (11) $$\begin{array}{r} w_{j}=\frac{g_{j}}{\overset{b}{\underset{j=1}{\sum }}g_{j}} \end{array}$$

The comprehensive score of the calculated results was used as a substitute variable for the integration degree of cultural tourism industry.

## Empirical Study

### Variable Selection

#### Explained Variable

This article uses the coupling coordination degree model to calculate the coupling coordination degree of 31 provinces and autonomous regions in China as the substitute variable CT of the integration level of cultural tourism industry.

#### Explaining Variable

The definition of digital economy by previous scholars mainly includes two aspects: in terms of form, digital economy is an economic form that guides and realizes the rapid optimal allocation and regeneration of resources and achieves high-quality economic development. At the technological level, digital economy is a collection of emerging technologies including big data, cloud computing, Internet of things, block chain, artificial intelligence, 5G communications, and so on. In the application level, its typical representatives are “new retail,” “new manufacturing,” and so on. According to the definition of digital economy, it is difficult to quantify the digital economy, so this article replaces the digital economy with the digital financial inclusion index used by Guo et al. (30). Digital financial inclusion index is a comprehensive definition based on the connotation and characteristics of the digital economy, and each index and dimension included should reflect a certain perspective of the overall digital economy [20]. See Table 3 for specific index system construction.

**Table 3:** Index system of digital economy.

**First-level dimension**	**Second-level dimension**	**Specific indicators**
		Number of Alipay accounts per 10,000 people
Coverage	Account coverage	Percentage of Alipay tied card users
		Average number of bank cards bound to each Alipay account
		Number of payments per person
	Payment transactions	Per capita payment
		Active users with high frequency (50 or more active times per year) accounted for 1 or more active times per year
		Per capita purchase of Yu 'ebao
	Money fund business	Per capita purchase amount of Yu 'ebao
		The number of people who buy Yu 'ebao per 10,000 Alipay users
		The number of users of Internet consumer loan in every ten thousand Alipay users
	Personal consumption loan	Number of loans per capita
		Per capita loan amount
		Number of users of Internet small and microbusiness loans per 10,000 adult Alipay users
Investment portfolio	Small and microoperator	Average number of loans for small and microbusiness owners
		Average loan amount of small and microbusiness operators
		Number of insured users per 10,000 Alipay users
	Insurance services	Number of insurance per capita
		Per capita insurance amount
		Number of people participating in Internet investment and financing per 10,000 Alipay users
	Investment business	Number of investments per capita
		Per capita investment
		Number of credit calls per natural person
	Credit business	Number of service users (including finance, accommodation, travel, social networking, etc.) using credit service per 10,000 Alipay users
	Mobility	Proportion of mobile payment transactions
		Proportion of mobile payment amount
	Affordable	Average loan interest rate for small and microbusiness operators
		Average personal lending rates
		Credibility
		Proportion of the payment amount of Huabei
Degree of digitization	Credibility	Proportion of free deposit Sesame Credit (compared with all cases requiring deposits)
		Proportion of Sesame Credit free deposit amount (compared with all cases requiring deposit)
		The proportion of users using QR code to pay
	Facilitation	The proportion of the amount paid by users using QR codes

The comprehensive score of digital economy of each province was calculated by entropy method as explanatory variable.

For COVID-19, the number of confirmed cases or deaths is generally used as explanatory variables in the literature on impact analysis. As a measure of epidemic severity, the number of confirmed COVID-19 cases in 31 provinces of China was selected as an explanatory variable.

#### Control Variable

In this article, urbanization level, industrialization level, foreign direct investment, and advanced industrial structure level are selected as control variables. The ratio of resident population to the total area of each region in the current year is used to indicate the local urbanization level; the number of industrial enterprises above designated size in each region is used to measure the local industrialization level; foreign direct investment to measure foreign direct investment; the ratio of the total output value of the tertiary industry and the total output value of the secondary industry measures the level of advanced industrial structure. Relevant data are from the statistical yearbook of each province.

### Model Specification

Based on the above selection of explanatory variables, explained variables, and control variables, this article establishes a multiple linear regression model to explore the direct impact of COVID-19 and digital economy on the integration of China's cultural tourism industry, and also the indirect impact of digital economy on the integration of China's cultural tourism industry during COVID-19. To avoid pseudoregression and eliminate heteroscedasticity in the model, each variable is processed logarithmically to obtain stable data without changing the nature and correlation of time series. The model is processed logarithmically as follows:


(1) $$\begin{array}{r} \ln\;CT_{it}=\alpha_{0}+\beta_{0}\ln\;DE_{it}+\beta_{1}\ln\;COV_{it}+\beta_{2}\ln\;SIZE_{it}\\ +\beta_{3}\ln\;CITY_{it}+\beta_{4}\ln\;FDI_{it}+\beta_{5}\ln\;ADV_{it}\\ +u_{i}+\zeta_{it} \end{array}$$

(2) $$\begin{array}{r} \ln\;CT_{it}=\alpha_{0}+\beta_{0}\ln\;DE_{it}+\beta_{1}\ln\;COV_{it}+\beta_{2}\ln\;DE_{it}*\ln\;COV_{it}\\ +\beta_{3}\ln\;SIZE_{it}+\beta_{4}\ln\;CITY_{it}+\beta_{5}\ln\;FDI_{it}\\ +\beta_{6}\ln\;ADV_{it}+u_{i}+\zeta_{it} \end{array}$$

Among them, CT represents integration of cultural tourism industry, DE represents digital economy, COV represents COVID-19, FDI represents foreign direct investment, SIZE represents industrialization level, CITY represents city size, and ADV represents advanced industrial structure. α_0_ is a constant, and ζ_*it*_ is a random error term.

### Data Sources and Descriptive Statistics

Descriptive statistics of each variable are shown in Table 4.

**Table 4:** Descriptive statistical results of each variable.

**Variables**	**Mean**	**Standard deviation**	**Min**	**Max**
Integration degree of cultural tourism industry	0.429	0.124	0.131	0.695
Digital economy (DE)	5.80	0.10	5.64	6.07
COVID-19 (COV)	4.26	3.34	0	11.13
Level of industrialization (SIZE)	1.834	1.197	−1.064	4.182
Urbanization level (CITY)	7.664	0.645	5.225	8.749
Foreign direct investment (FDI)	3.258	0.847	1.450	6.194
Advanced industrial structure (ADV)	4.624	0.580	3.230	6.193

According to the descriptive statistics in Table 3, the average integration degree of cultural tourism industry is 0.429, and the maximum and minimum values are 0.695 and 0.131, respectively. It can be found that the integration level of China's cultural tourism industry is relatively balanced. The average value of digital economy is 5.8, the maximum and minimum values are 6.07 and 5.64, respectively, which indicates that the overall level of digital economy in China is relatively balanced, and there is little difference in investment in digital economy among different regions. The COVID-19 outbreak average was 4.26, and the maximum and minimum values were 11.13 and 0, respectively. With the exception of Hubei province, there is little difference in the number of confirmed COVID-19 cases among provinces and autonomous regions.

According to the descriptive statistical results of control variables, the average value of industrialization level is 1.834, and the maximum and minimum values are 4.182 and −1.064, respectively, indicating that industry in some regions of China has been seriously affected during the epidemic. The average value of urbanization level is 7.664, the maximum and minimum values are 8.749 and 5.225, respectively, indicating that China's urbanization level is relatively balanced. The average value of foreign direct investment is 3.258, the maximum and minimum values are 8.749 and 5.225, respectively, indicating that China's overall level of attracting foreign investment is relatively strong. The average value of industrial structure upgrading is 4.624, and the maximum and minimum values are 6.193 and 3.230, respectively, indicating that the production scale and technological level of industries in some regions are still at a low level.

## Positive Economics

### The Direct Impact of COVID-19 and Digital Economy on the Integration of China's Cultural Tourism Industry

Table 5 shows the influence of COVID-19 and digital economy on the integration of cultural tourism in China in Equation (1). The coefficient of digital economy is significantly positive at the level of 1% and shows an increasing trend, indicating that the development of digital economy during the COVID-19 epidemic has indeed promoted the integrated development of China's cultural tourism industry, and the coefficient of digital economy is larger than that in 2019, the initial stage of the epidemic. The coefficient of COVID-19 is significantly negative at the 1% level, with an increasing trend all the time. This indicates that with the outbreak of COVID-19 in Wuhan in China, it is spreading rapidly to the whole country, and the negative impact on the integration of culture and tourism in China is increasingly strong. This verifies hypothesis H1. The coefficient of the observed square term of COVID-19 is significantly positive at the level of 1%. It shows that COVID-19 has a long-term effect on the integration of China's cultural tourism industry, which presents a U-shaped trend that will promote the integration of cultural tourism industry in the long run, which verifies hypothesis H2.

**Table 5:** The direct impact of COVID-19 and digital economy on the integration of China's cultural tourism industry.

	**2019**	**2020**
Digital economy (DE)	0.2451*** (2.42)	0.6251*** (3.23)
COVID-19 (COV)	−0.0251*** (−0.24)	−0.1256*** (−0.75)
(COV^2^)	0.0145*** (0.41)	0.1521*** (0.62)
Level of industrialization (SIZE)	1.4152*** (5.23)	1.5124*** (5.67)
Urbanization level (CITY)	0.2524*** (2.34)	0.1641*** (2.17)
Foreign direct investment (FDI)	−0.153* (−1.21)	−0.2231* (−1.64)
Advanced industrial structure (ADV)	1.5124* (6.21)	2.2241* (6.45)
Constant	−3.5355*** (−8.55)	−4.5524*** (−8.96)

***, ** and * represent level of significance at 1%, 5% and 10%.

Observing the results of the control variables, it can be seen that the coefficient of industrialization level is significantly positive at the level of 1%, and the promoting effect is significant. The coefficient of urbanization level is significantly positive at the level of 1%, which may be because with the improvement in infrastructure, a solid foundation has been laid for the integration of cultural tourism industry. Foreign direct investment is significantly negative at the level of 10%, which may be due to crowding out the level of domestic investment, the decline of the overall level of domestic investment, and the lack of innovation ability, which also hinders the integrated development of China's cultural tourism industry. The industrialization level is significantly positive at 1% level, which also indicates that the integration of China's cultural tourism industry cannot be separated from the development of industrialization. Under the impact of COVID-19, the industry has been hit hard, and there is an urgent need to develop digital economy, make the industry to intelligent transformation, and promote the integration of China's cultural tourism industry.

### Indirect Impact of COVID-19 and Digital Economy on the Level of Integration of China's Cultural Tourism Industry

Table 6 shows the indirect impact of COVID-19 epidemic and digital economy on the integration of China's cultural tourism industry in Equation (2). In this result, the crossterm of COVID-19 epidemic and digital economy is focused, and it can be found that the crossterm of COVID-19 epidemic and digital economy is significantly positive at the level of 1% and presents an increasing trend. On the one hand, it proves that the development of digital economy during COVID-19 has promoted the integration of China's cultural tourism industry; on the other hand, it proves that the positive effect of digital economy on the integration of China's cultural tourism industry during the COVID-19 pandemic is gradually strengthened compared to previous ones. By observing the coefficients of COVID-19 and digital economy, it can be seen that, by comparing the coefficients of COVID-19 and digital economy in Table 5, the significance of the coefficients of explanatory variables in Table 6 remains unchanged, but the coefficients decrease slightly, which also proves that digital economy plays an intermediary role in the process of COVID-19 affecting the integration of China's cultural tourism industry.

**Table 6:** Indirect impact of COVID-19 and digital economy on the integration of China's cultural tourism industry.

	**2019**	**2020**
Digital economy (DE)	0.1241*** (1.56)	0.3156*** (2.02)
COVID-19 (COV)	−0.0102*** (−0.09)	−0.1721*** (−0.39)
(COV^2^)	0.0131*** (0.51)	0.1251*** (0.59)
(DE*COV)	0.4512*** (3.51)	1.6521*** (7.02)
Level of industrialization (SIZE)	1.4241*** (5.21)	1.5244*** (5.52)
Urbanization level (CITY)	0.2212*** (2.22)	0.1241*** (2.08)
Foreign direct investment (FDI)	−0.1241* (−1.09)	−0.2241* (−1.32)
Advanced industrial structure (ADV)	1.5553* (6.11)	2.2767* (6.34)
Constant	−3.5685*** (−8.12)	−4.5457*** (−8.24)

***, ** and * represent level of significance at 1%, 5% and 10%.

By observing the coefficients of the control variables, it can be seen that on the premise of invariable significance, the coefficients are all reduced to varying degrees. This shows that the impact of COVID-19 on the digital economy reduces the impact of other variables on the integration of cultural tourism industry. This further illustrates the importance of the digital economy to restore the integration of China's cultural tourism industry during the COVID-19 pandemic.

## Conclusions and Policy Recommendations

### Conclusions

Through empirical research on the direct impact of COVID-19 and digital economy on the integration of China's cultural tourism industry, it is found that the development of digital economy during the COVID-19 pandemic has indeed promoted the integration of China's cultural tourism industry, and the digital economy has promoted the integration of China's cultural tourism industry more strongly than in 2019, the initial stage of the epidemic. With the rapid spread of COVID-19 to the whole country after the outbreak of COVID-19 in Wuhan, the negative impact on the integration of China's cultural tourism industry is also increasingly strong. Moreover, COVID-19 has a long-term effect on the integration of China's cultural tourism industry, showing a U-shaped trend, which will promote the integration of cultural tourism industry in the long run.

An empirical study on the indirect impact of COVID-19 and digital economy on the integration of China's cultural tourism industry found that the development of digital economy during COVID-19 has promoted the integration of China's cultural tourism industry, and the positive effect of digital economy on the integration of China's cultural tourism industry has gradually strengthened compared to the previous situation. The digital economy has played a mediating role in the impact of COVID-19 on the integration of China's cultural tourism industry.

### Policy Recommendations

In view of the conclusions drawn in this article, the following policy recommendations are put forward:

(1) Formulating macropolicies: it is necessary to control the domestic and international trends of the epidemic development, steadily and comprehensively promote the work of domestic economic and social development in the postepidemic period, pay close attention to the development of the global epidemic, and actively plan countermeasures. The central government should formulate policies for the integrated development of China's cultural tourism industry after the epidemic and fully consider the long-term and short-term impacts of COVID-19 on the integration of China's cultural tourism industry. We will give full play to the leading role of fiscal policies. The Ministry of Finance and other departments will jointly put forward supportive policies for stabilizing economic and social development, and relevant departments will cooperate in their implementation. Financial departments at all levels should give full play to the financial advantages of all levels and local governments to provide special interest loans to key epidemic prevention and control enterprises.

(2) Formulating policies related to the digital economy: transform the effective short-term digital economy pilot policies into long-term policies, take “digital industrialization, industrial digitization, and digital governance” as the main line of development, accelerate the upgrading of provincial guidance on accelerating the development of digital economy, and promote the implementation of pilot policies conducive to the development of digital economy. The epidemic has further highlighted the importance and vulnerability of small and medium-size enterprise in China's employment and economic development. Some short-term policies issued by governments at all levels to support the development of small- and medium-sized digital enterprises are considered to be timely and effective after postevaluation and can be transformed into long-term policies based on the coordination and improvement.

(3) To strengthen the capacity of the digital economy to cope with risks: First, in response to the shortcomings exposed in the epidemic response, big data, artificial intelligence, blockchain, smart supply chain, and other technologies are used to enable emergency prevention and control of major public health events and effectively improve the effectiveness of major epidemic prevention and control. Second, we will systematically identify weaknesses in the national reserve system, improve reserve efficiency, optimize the distribution of production capacity of key supplies, and improve contingency plans. Third, based on the vulnerability of the public health governance system exposed by the epidemic, we will comprehensively and thoroughly investigate and rectify similar problems existing in the governance system in other fields, enhance the building of emergency response capacity supported by digital technology, and further establish and improve China's modern public security emergency management system.

(4) Improve the digital system: strengthen talent support, intensify efforts to tackle cutting-edge technologies and train cutting-edge talents, and accelerate the improvement in strategic scientific and technological forces and strategic reserve capacity in the field of epidemic prevention and control and public health. Second, we will accelerate the development of a platform support system for the development of the digital economy. From both the supply side and the demand side, accelerate the construction and promotion of industrial Internet platform and the popularization of the application of industrial Internet for small- and medium-sized enterprises, form a multilevel and systematic platform development system, and promote the connection of all elements of industry and optimal allocation of resources. Third, we will accelerate the improvement of policy and institutional support systems for the development of the digital economy. We will establish and improve policies and regulations that suit the development of digital economy industries, further deepen reform to delegate power, improve regulation and services to promote continuous improvement of the business environment, strengthen oversight over digital transactions, and improve data security systems.

## Data Availability Statement

The raw data supporting the conclusions of this article will be made available by the authors, without undue reservation.

## Author Contributions

Material preparation, data collection, and analysis were performed by XLi and XLia. The first draft of the manuscript was written by XLi. All authors commented on previous versions of the manuscript, contributed to the study conception and design, read, and approved the final manuscript.

## Conflict of Interest

The authors declare that the research was conducted in the absence of any commercial or financial relationships that could be construed as a potential conflict of interest.

## Publisher's Note

All claims expressed in this article are solely those of the authors and do not necessarily represent those of their affiliated organizations, or those of the publisher, the editors and the reviewers. Any product that may be evaluated in this article, or claim that may be made by its manufacturer, is not guaranteed or endorsed by the publisher.
